# Development of a Multiplexed Electrochemical Aptasensor for the Detection of Cyanotoxins

**DOI:** 10.3390/bios14060268

**Published:** 2024-05-24

**Authors:** Amina Rhouati, Mohammed Zourob

**Affiliations:** 1 Department of Chemistry, Alfaisal University, Al Zahrawi Street, Al Maather, Al Takhassusi Road, Riyadh 11533, Saudi Arabia; arhouati@alfaisal.edu; 2 Bioengineering Laboratory, Higher National School of Biotechnology, Constantine 25100, Algeria

**Keywords:** multiplexed detection, aptasensor, electrochemical, cyanotoxins, freshwater contaminants

## Abstract

In this study, we report a multiplexed platform for the simultaneous determination of five marine toxins. The proposed biosensor is based on a disposable electrical printed (DEP) microarray composed of eight individually addressable carbon electrodes. The electrodeposition of gold nanoparticles on the carbon surface offers high conductivity and enlarges the electroactive area. The immobilization of thiolated aptamers on the AuNP-decorated carbon electrodes provides a stable, well-orientated and organized binary self-assembled monolayer for sensitive and accurate detection. A simple electrochemical multiplexed aptasensor based on AuNPs was designed to synchronously detect multiple cyanotoxins, namely, microcystin-LR (MC-LR), Cylindrospermopsin (CYL), anatoxin-α, saxitoxin and okadaic acid (OA). The choice of the five toxins was based on their widespread presence and toxicity to aquatic ecosystems and humans. Taking advantage of the conformational change of the aptamers upon target binding, cyanotoxin detection was achieved by monitoring the resulting electron transfer increase by square-wave voltammetry. Under the optimal conditions, the linear range of the proposed aptasensor was estimated to be from 0.018 nM to 200 nM for all the toxins, except for MC-LR where detection was possible within the range of 0.073 to 150 nM. Excellent sensitivity was achieved with the limits of detection of 0.0033, 0.0045, 0.0034, 0.0053 and 0.0048 nM for MC-LR, CYL, anatoxin-α, saxitoxin and OA, respectively. Selectivity studies were performed to show the absence of cross-reactivity between the five analytes. Finally, the application of the multiplexed aptasensor to tap water samples revealed very good agreement with the calibration curves obtained in buffer. This simple and accurate multiplexed platform could open the window for the simultaneous detection of multiple pollutants in different matrices.

## 1. Introduction

The overgrowth of cyanobacteria, also known as harmful algal cyanobacterial blooms, occurs under a combination of certain environmental factors such as nutrient levels, water temperature, and salinity [1]. Recently, this phenomenon has raised major concerns worldwide, mainly because of the water supply contaminations it causes. Water can be contaminated by cyanobacteria or their metabolites, which are known as cyanotoxins [2]. It has been reported that 50% of algal blooms can contain harmful toxins to aquatic ecosystems, plants, animals and human health [3]. More than a hundred cyanotoxins with diverse chemistry and toxicity have been identified. According to their targeted organ, they are categorized into three classes: hepatotoxic, neurotoxic and dermatotoxic. Based on their chemical structure, the major types of cyanotoxins are cyclic peptides, alkaloids and lipopolysaccharides [3].

Hepatotoxic microcystins (MCs) are the most frequent class of cyanotoxins occurring in aquatic ecosystems and drinking water. Among more than 85 identified variants, microcystin-LR is the most commonly found and the most toxic one throughout the world [4]. It is a cyclic heptapeptide (Leucine/Arginine variant), reported as an inhibitor of protein serine/threonine phosphatases 2A and 1 that may lead to hepatic ultrastructure damage and certain cancers even at low concentrations [5,6]. For that reason, the World Health Organization (WHO) have established a guideline of 1 µg/L as a maximum level for MC-LR in drinking water [7]. Besides MCs, Cylindrospermopsin (CYL) is gaining importance because of its expansion to temperate zones, such as Germany and France, after being firstly identified in tropical zones [8]. Moreover, besides its hepatoxic effects, CYL is now considered cytotoxic and genotoxic [9]. Structurally, CYL is a tricyclic alkaloid that comprises a guanidine and an uracil moiety potentially responsible for its toxicity [10]. Due to its harmful effects, a maximum level of 1 µg/L was suggested for CYL in drinking water [11].

Neurotoxins have also gained much attention because of their effects on the nervous system. Saxitoxins and anatoxins are the most studied ones; they are both alkaloids, which are known for their physiological effects interfering with nerve cells [12]. It has been reported that saxitoxins block sodium ion channels in the nerve axon membrane and induce nerve dysfunction, paralysis and even death in humans [13]. Anatoxin-a acts by mimicking acethylcholine and binds to its receptors, leading to various animal poisoning incidents [13]. However, no guidelines have been established for these two toxins since no intoxication through drinking water has been demonstrated, but 3 µg/L has been suggested [14].

Okadaic acid (OA) and its related compounds, dinophysistoxins, constitute a structural complex family of thermostable lipophilic polyether compounds responsible for human diarrhetic shellfish poisoning [15]. This group of marine toxins is more abundant in southern Europe, where the maximum regulatory limit of OA in shellfish was set at 160 μg/kg [16].

In general, certified laboratories use traditional methods like chromatographic techniques, mass spectrometry and biological assays for cyanotoxin monitoring. However, these techniques are expensive, requiring sophisticated equipment and qualified staff. In addition, they are not suitable for in situ monitoring, which is highly desired to minimize the risk to human health through exposure to cyanotoxins [17]. Recently, several reports have described different biosensing techniques based on enzymes, antibodies and aptamers to detect marine toxins with lower cost and at low levels. Biosensors can be easily miniaturized to allow simple and real-time cyanotoxin analysis [18]. Among the different types that have been reported, affinity-based electrochemical biosensors have gained much attention. Whether based on antibodies or aptamers, this class of biosensors provides accurate, specific and sensitive detection [19]. Recently, DNA-based biosensors have shown great progress [20,21]. Aptamers have rivaled antibodies for the advantages they offer. Aptamers are short sequences of oligonucleotides that are synthesized in vitro via a low-cost and simple chemical process called SELEX (Systematic Evolution of Ligands by Exponential Enrichment). Therefore, they are more stable than antibodies and do not suffer from batch-to-batch variability [22]. Moreover, they can be labeled, allowing their application in optical and electrochemical detection. Furthermore, aptamers can be used in a label-free mode, given the conformational change they undergo after target binding [23].

Several schemes of electrochemical aptasensors have been proposed for cyanotoxin detection [24,25]. For instance, specific aptamers to different cyanotoxins have been previously selected by our group. They have been successfully applied in the electrochemical sensing of MC-LR [26], CYL [27], anatoxin-α [28] and okadaic acid [29]. Zheng et al. developed a saxitoxin-binding aptamer [30], which was subsequently applied in electrochemical aptasensing [31,32]. The reported aptasensors exhibited high sensitivity, where the detection limits were below the limits established by the WHO. However, they were designed to detect only one toxin, while cyanobacterial blooms may contain complex mixtures of several classes of cyanotoxins. Therefore, there is an urgent need to develop reliable biosensors to detect different cyanotoxins on the same chip.

To the best of our knowledge, no multiplexed aptasensor has been developed for the simultaneous detection of cyanotoxins. Herein, we constructed an electrochemical multiplexed platform by immobilizing five thiolated aptamers specific to MC-LR, CYL, anatoxin-α, saxitoxin and OA on electrical printed carbon array electrodes modified with gold nanoparticles (AuNPs). Then, the synchronous detection of the mixture of cyanotoxins was performed by square-wave voltammetry (SWV). The multi-analyte approach was based on a single-array system to produce distinguishable electrochemical signals, differentiating the corresponding targets. Satisfactory results were obtained; the aptasensor showed high sensitivity, good reproducibility and selectivity with an excellent applicability in spiked tap water samples. The proposed strategy meets the need for simultaneous monitoring of freshwater contaminants to prevent human fatalities caused by cyanobacterial toxins.

## 2. Materials and Methods

### 2.1. Chemicals and Instrumentation

HPLC-purified thiolated aptamers were provided from Metabion International (Planegg, Germany). The sequences are presented in Table 1. The DNA oligonucleotides were dissolved in ultrapure Milli-Q water to make the stock solutions and stored at −20 °C until further use. The DNA solutions used in the experiments were diluted with a binding buffer. Microcystin-LR, Cylindrospermopsin, anatoxin-α, saxitoxin and okadaic acid sodium salt were purchased from Enzo Life Sciences (Toronto, ON, Canada). The aptamer and toxin solutions used in the experiments were diluted with the binding buffer (50 mM Tris, pH 7.5, 150 mM NaCl, and 2 mM MgCl_2_). Potassium ferrocyanide [K_4_Fe(CN)_6_], potassium ferricyanide [K_3_Fe(CN)_6_], phosphate-buffered saline (PBS), gold (III) chloride solution, potassium nitrate (KNO_3_), bovine serum albumin (BSA) and potassium nitrate (KNO_3_) were purchased from Sigma-Aldrich, Darmstadt, Germany.

All the electrochemical measurements were performed by a multichannel AUTOLAB potentiostat PGSTAT302N obtained from Metrohm, Netherlands. The potentiostat was connected to a personal computer and operated by the Nova 1.11 software. The sensors were fabricated on disposable electrical printed (DEP) microarray electrodes obtained from BioDevice Technology (Nomi, Japan). The array electrode was composed of a 3-electrode configuration with eight individually addressable carbon working electrodes (round shape), a ring-shaped carbon counter electrode and a central silver/silver chloride reference electrode. The sensor connector employed to connect the DEP electrodes to the potentiostat was obtained from BioDevice Technology.

### 2.2. Fabrication of the Multiplexed Aptasensor and Analytical Experiments

The multiplexed aptasensor was fabricated by functionalizing each electrode in the microarray with one aptamer. For that, AuNPs were electrodeposited on the eight-carbon electrodes by covering the surface chip with a solution of 100 μL of 8 mM HAuCL_4_ and applying twenty CV scans from −0.2 to −1.2 V at 50 mV/s. After that, five of the eight electrodes were incubated with 1 µM of the thiolated aptamers specific to the five cyanotoxins (MC-LR, CYL, anatoxin-α, saxitoxin and OA). After overnight incubation, non-specific binding sites were blocked with 1% BSA for 40 min at room temperature. Finally, the multiplexed sensing chip was stored at 4 °C for further use.

Cyclic voltammetry (CV) at a scan rate of 100 mV s^−1^ within a potential range of −0.6 to 0.8 V was used for the characterization of the biosensor fabrication steps in a 5 mM [K_4_Fe(CN)_6_]/[K_3_Fe(CN)_6_] redox solution. Electrochemical impedance spectroscopy (EIS) was used to record within a frequency range of 10,000 to 0.1 Hz, an AC amplitude of 10 mV and a DC potential of +0.20 V.

For the biosensing experiments, 1 μL of each toxin solution was dropped on its specific electrode on the chip and incubated for 20 min at room temperature. Diverse amounts of each toxin ranging from 0 to 200 nM, which had been prepared in the binding buffer, were tested. After rinsing the surface with PBS, electrochemical measurements were performed of the redox couple. The SWV measurements were recorded at an interval time of 0.04 s, a frequency of 25 Hz, a scan rate of 125 mV s^−1^, an amplitude of 20 mV and a step potential of −5 mV.

### 2.3. Interference Study

To assess the specificity of the proposed multiplexed aptasensor, each aptamer-modified surface was incubated with 1 μL of its specific target and the other four non-specific toxins, separately. For example, the electrode functionalized with the MC-LR aptamer was incubated with MC-LR as well as Cyl, anatoxin, saxitoxin and okadaic acid at the same concentration of 100 nM. Then, the aptasensor responses to the different toxins were electrochemically recorded and compared by determining the change in the peak current after the binding.

### 2.4. Biosensing Experiments in Tap Water Samples

To demonstrate the applicability of the multiplexed aptasensor array, tap water samples were collected and spiked with the five different toxins. Then, the presence of the targeted cyanotoxin was investigated electrochemically using the same conditions.

## 3. Results and Discussion

### 3.1. Mechanism of the Multiplexed Electrochemical Aptasensor

**Scheme 1:**
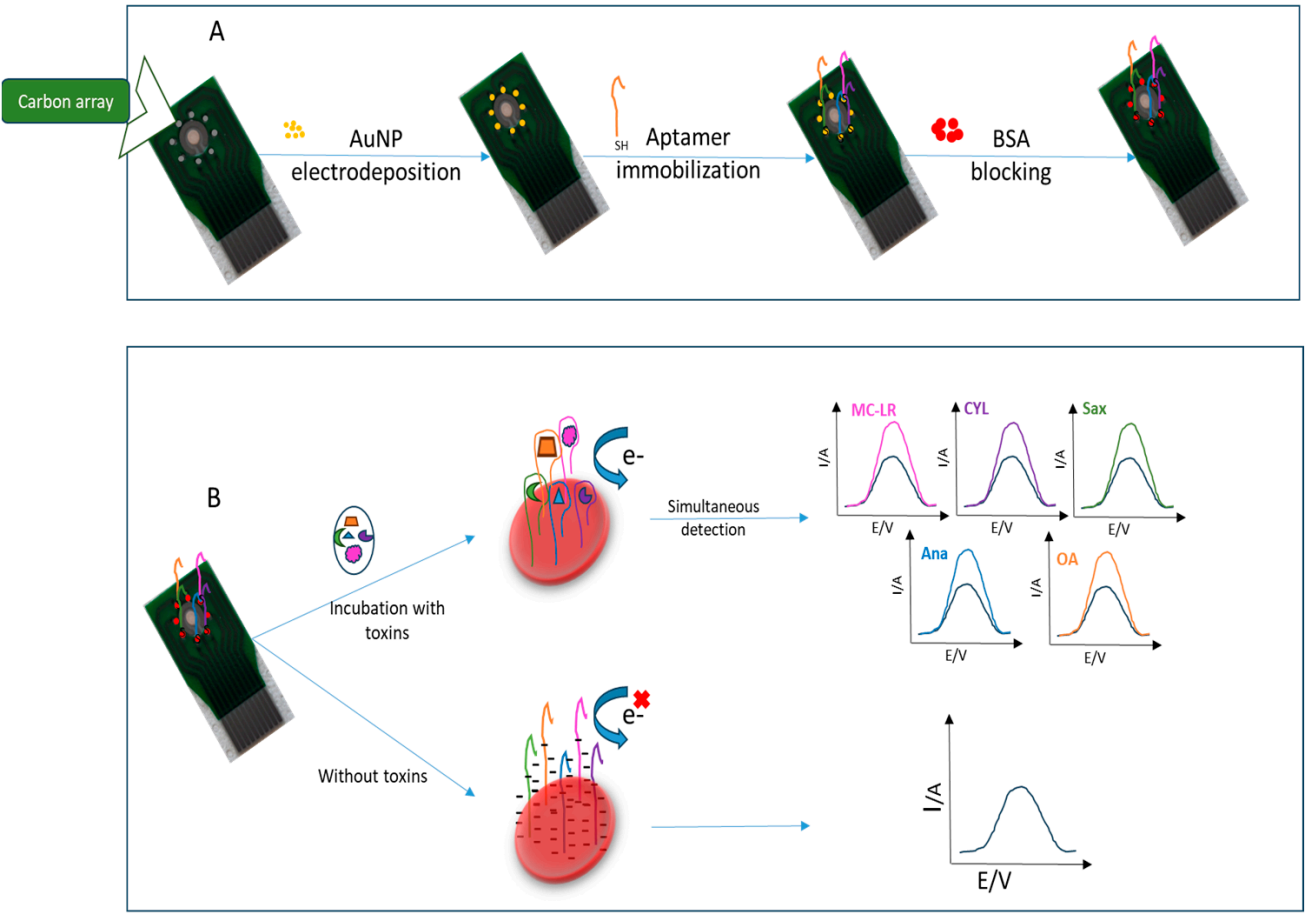
Schematic illustration of the fabrication steps (**A**) and the working principle (**B**) of the multiplexed cyanotoxin aptasensor.

Scheme 1 depicts the fabrication steps as well as the working principle of the multi-toxin aptasensor. As mentioned above, the proposed aptasensor was constructed on a chip with eight screen-printed carbon electrodes. The carbon surface was modified with gold nanoparticles via electrodeposition based on the procedure described in the experimental section. Then, the five thiolated aptamers specific to the targeted toxins (MC-LR, CYL, anatoxin-α, saxitoxin and OA) were immobilized via thiol covalent attachment to the gold layer. It has been demonstrated that thiols exhibit high adsorption on gold, where the sulfur groups spontaneously adsorb onto the metal surface. This phenomenon is mainly based on diffusion-controlled physisorption and chemisorption of the thiolated DNA on the gold surface, followed by the crystallization process [33]. In parallel, given their small size and versatility, aptamers allow efficient immobilization in high-density monolayers. Therefore, a stable, well-orientated and organized binary self-assembled monolayer is formed, resulting in a higher aptamer density, improved accessibility for analyte detection and lower sample-to-sample variability [33]. The aptamers used in this work have been shown in previous reports to have excellent binding to their targets with remarkable sensitivity and selectivity [26,27,28,29,30]. Herein, the multi-analyte detection principle is based on the electrochemical monitoring of the specific recognition between the immobilized aptamers and their targets by square-wave voltammetry. The formation of the aptamer–toxin complex leads to a change in the voltametric signal. In the absence of the targets, the negatively charged backbone of DNA hampers electron transfer to the electrode surface, resulting in a lower reduction peak current of the redox couple. In the presence of the targeted toxins, each analyte will be specifically bound to its corresponding aptamer-functionalized electrode in the multiplexed array. It is well known that the binding of aptamers induces a conformational change of DNA to a three-dimensional structure. Subsequently, the number of negative charges decreases, thus allowing electron transfer to the surface. Therefore, the voltametric peak current of ferro/ferricyanide will increase by increasing the cyanotoxin concentration. The peak variations provide an accurate strategy for the simultaneous quantification of our five analytes (MC-LR, CYL, anatoxin-α, saxitoxin and OA).

### 3.2. Characterization of the Aptasensor Fabrication Strategy

The different fabrication steps of the multiplexed aptasensor were characterized electrochemically using cyclic voltammetry (CV). This is a powerful and popular electrochemical technique commonly employed to investigate reduction and oxidation processes. Any phenomenon occurring on the working electrode can be followed by evaluating the characteristic parameters derived from the cyclic voltammograms, including the oxidation and reduction peak potentials, peak-to-peak separation and peak currents [34]. With the successive modification steps of the surface and the immobilization of the aptamers, electron transfer is more/less hampered, resulting in a decrease/increase in the current peaks, thus allowing the characterization of different events involved in the biosensor construction.

**Figure 1:**
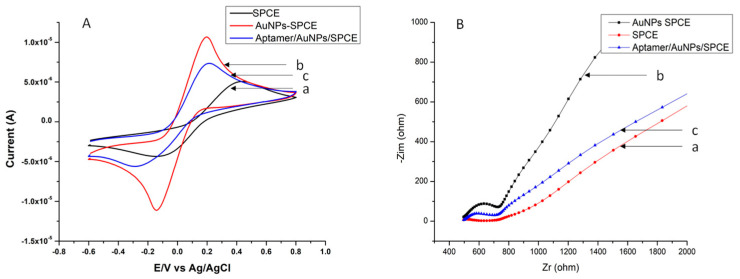
(**A**) Cyclic voltammograms corresponding to each step of the multiplexed aptasensor fabrication process, obtained by scanning the potential between −0.6 V and 0.8 V with a scan rate of 50 mV/s: (a) bare carbon electrode, (b) AuNP-modified carbon electrodes and (c) aptamer/AuNP/carbon electrodes. (**B**) Impedance spectra obtained over the frequency range from 10 KHz to 0.1 Hz. All the electrochemical measurements were recorded in a 5 mM [Fe(CN)_6_]^−4/−3^ redox couple in PBS at a pH of 7.4.

Herein, cyclic voltammograms were recorded in the presence of the reversible [K_4_Fe(CN)_6_]/[K_3_Fe(CN)_6_] redox system by scanning the potential between −0.6 V and 0.8 V, with a scan rate of 50 mV s^−1^. Characterization of each one of the five electrodes comprising the multiplexed aptasensing array was performed. Figure 1A shows the obtained voltammograms for one of them: the bare electrode (a), AuNPs-SPCE (b) and aptamer–AuNP−SPCE surface (c). First, curve a shows that the [Fe(CN)_6_]^3/4−^ redox couple exhibited a characteristic quasi-reversible voltammogram on the bare carbon electrode with well-defined oxidation and reduction peaks *ip* (and a peak-to-peak separation of 0.57 V). Then, a dramatic increase in the anodic and cathodic peak currents was observed after the gold nanoparticle deposition, accompanied by a decrease in the peak-to-peak separation to 0.33 V (curve b). This phenomenon can be attributed to the high electronic conductivity of gold nanoparticles, which leads to a large electroactive area, thus enhancing the electron transfer rate. The high surface-to-volume ratio and high surface energy of AuNPs enhance the electron transfer between the redox probe [K_4_Fe(CN)_6_]/[K_3_Fe(CN)_6_] and the electrode surface [35]. Finally, after the immobilization of the aptamer, we noted that the electrochemical reaction was inhibited, where the peak current *ip* decreased and the peak-to-peak separation (ΔEp) increased to 0.51 V. The self-assembly of the thiolated aptamers on the AuNP-modified surface hindered the access of redox molecules, mainly due to the electrostatic repulsion between the negatively charged backbone of DNA and the redox couple anions. Therefore, a decrease in the electron transfer was observed. These results are in good agreement with previous reports which demonstrated the effect of aptamer immobilization on electron transfer [34]. These results are also in good agreement with the impedance spectra shown in Figure 1B. The Niquist diagrams of the AuNP-modified SPCEs showed a low resistance of the surface because of the excellent electrochemical activity of gold nanoparticles. Then, after adding the aptamer solution, the surface resistance increased, showing the successful covalent binding between the thiol groups and the gold nanoparticles.

### 3.3. Optimization of the Incubation Time

**Figure 2:**
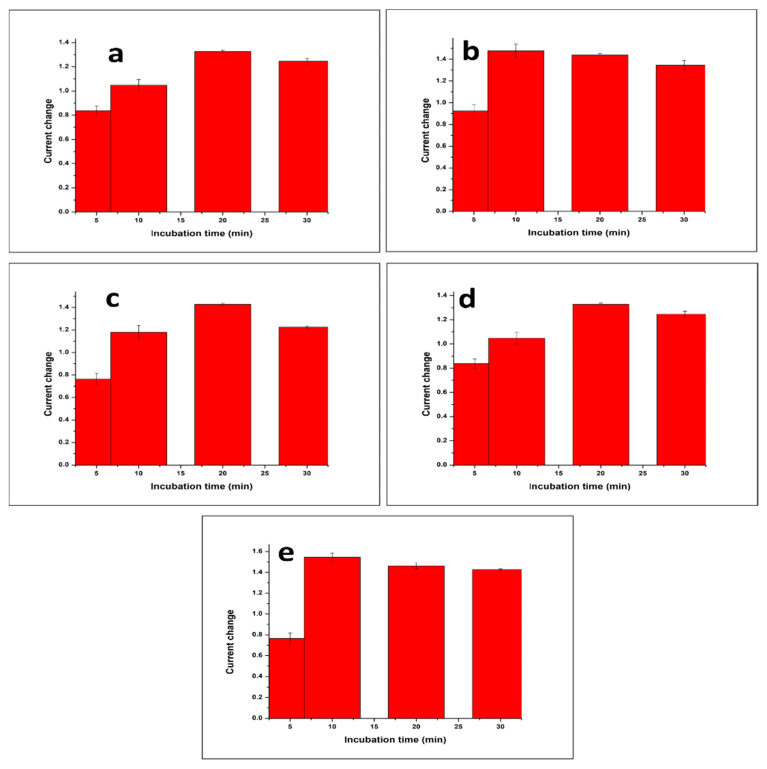
Effect of incubation time on the multiplexed aptasensor response (SWV current change) at a concentration of 100 nM of MC-LR (**a**), CYL (**b**), anatoxin-α (**c**), saxitoxin (**d**) and OA (**e**). The SWV measurements were recorded at an interval time of 0.04 s, a frequency of 25 Hz, a scan rate of 125 mV s^−1^, an amplitude of 20 mV and a step potential of 5 mV in 5 mM of [K_4_Fe(CN)_6_]/[K_3_Fe(CN)_6_]. The current change was calculated as *i*^0^ − *i*, where *i*^0^ and *i* correspond to the current peak before and after incubation with the target for different durations.

The incubation time of an aptamer with its analyte plays a key role in the performance of an aptasensor. We studied the effect of this factor on the electrochemical response of our multiplexed array. Each electrode was incubated with a fixed concentration of its specific analyte for different periods varying from 5 to 30 min. The array aptasensor responses were calculated by determining the current peak change after binding with the cyanotoxins. Figure 2a–e show the relation between the biosensor responses and the incubation time for MC-LR, CYL, anatoxin-α, saxitoxin and OA, respectively. For the OA and CYL aptasensors, the best response was obtained after 10 min of incubation. After that, no significant increase was noted. For saxitoxin and anatoxin, the signal increased until 20 min. After that, the electrochemical response started to decrease. However, the electrochemical response clearly increased with increasing incubation time for microcystin, but the increase after 20 min was not significant. Therefore, 20 min was chosen as the optimal recognition time in the subsequent experiments. Triplicate measurements were performed for all tests.

### 3.4. Analytical Performance of the Multiplexed Aptasensor

#### 3.4.1. Electrochemical Response for Multi-Toxin Detection

**Figure 3:**
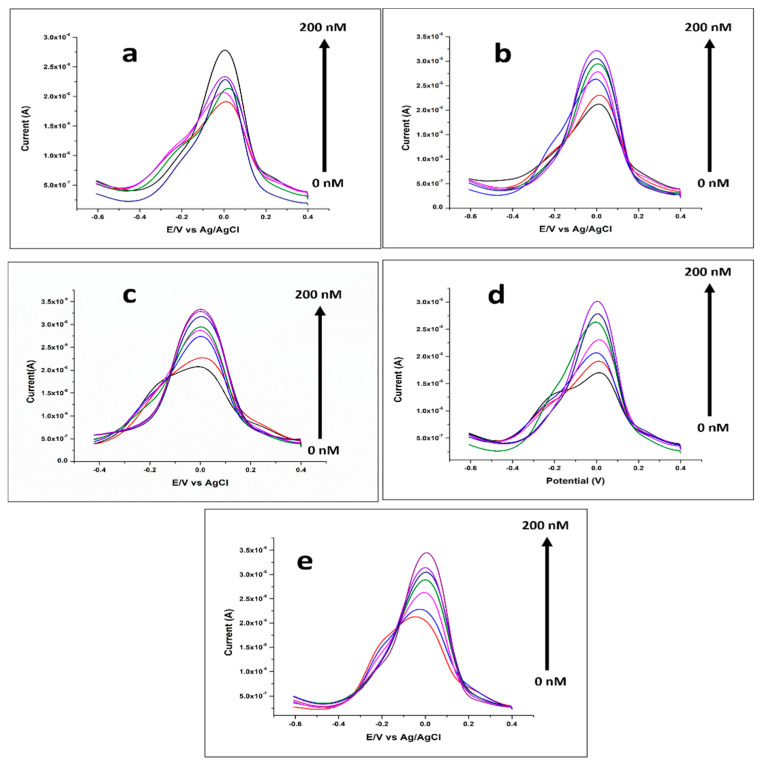
SWV responses of the proposed multiplexed aptasensor after incubation with various concentrations of MC-LR (**a**), CYL (**b**), anatoxin-α (**c**), saxitoxin (**d**) and OA (**e**). The SWV measurements were recorded at an interval time of 0.04 s, a frequency of 25 Hz, a scan rate of 125 mV s^−1^, an amplitude of 20 mV and a step potential of 5 mV in 5 mM of [K_4_Fe(CN)_6_]/[K_3_Fe(CN)_6_].

To study the response of the multiplexed aptasensing platform at different concentrations of the targeted cyanotoxins, each functionalized electrode was incubated with increasing amounts of the corresponding analyte. For that, the proposed array was incubated with MC-LR, CYL, anatoxin-α, saxitoxin and OA in the range of 18 pM to 200 nM for 20 min. Then, multi-toxin recognition by their specific aptamers was studied by monitoring the reduction of the [Fe(CN)_6_]^3/4−^ redox system using SWV. Figure 3a–e show the obtained voltammograms for the five aptamer-functionalized electrodes before and after incubation with the toxins within the mentioned range of concentrations. As can be seen, the formation of the aptamer–toxin complex on the five electrodes of the multiplexed array induces a change in the voltametric signal as registered by an increase in the SWV peak currents (*ip*). We noted that the reduction peak clearly increased with increasing target concentration until the concentration of 200 nM. The observed behavior is in good agreement with previous aptasensors developed for cyanotoxin electrochemical sensing [25,32]. This is commonly explained by the conformational change of the aptamer upon toxin binding, inducing an increase in electron transfer.

#### 3.4.2. Calibration Curves and Sensitivity

**Figure 4:**
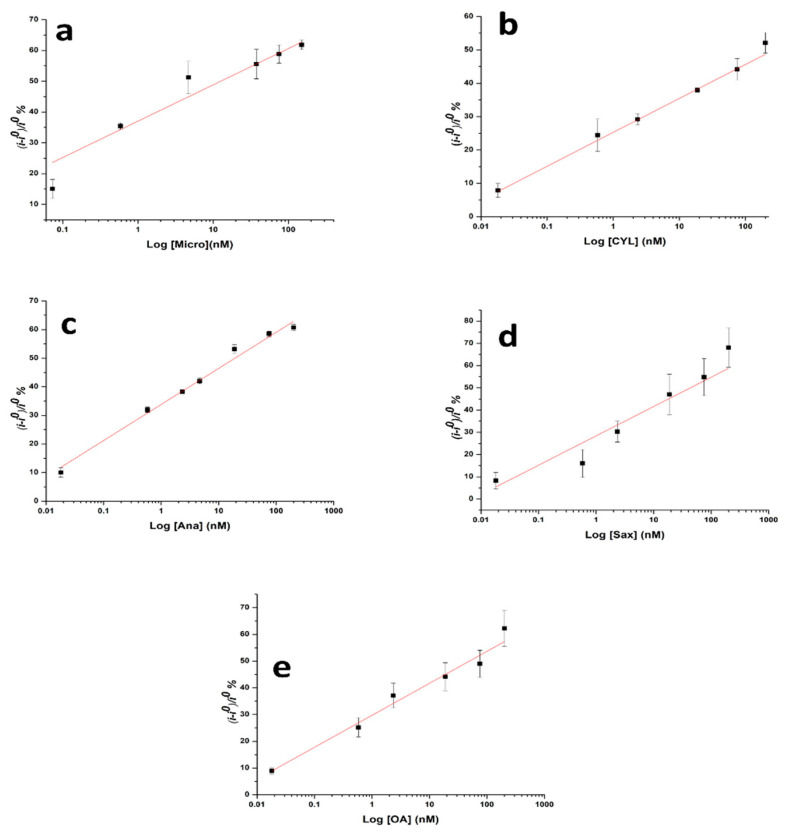
Calibration curves of the multiplexed aptasensor toward MC-LR (**a**), CYL (**b**), anatoxin-α (**c**), saxitoxin (**d**) and OA (**e**) in a binding buffer (pH 7.4) solution. Plots of the aptasensor responses ((*i*^0^ − *i/i*^0^) *%*) versus logarithm of toxin concentration (nM). The SWV measurements were recorded at an interval time of 0.04 s, a frequency of 25 Hz, a scan rate of 125 mV s^−1^, an amplitude of 20 mV and a step potential of −5 mV in 5 mM of [K_4_Fe(CN)_6_]/[K_3_Fe(CN)_6_]. Triplicate measurements were performed for all tests.

The obtained responses for each aptasensor were used to plot five calibration curves at varying concentrations of the targeted toxins. For that, the reduction peak current decrease rate ((*i* − *i*^0^)*/i*^0^ *%*) was calculated for each modified carbon electrode array (SPCE/AuNPs/APT) before (*i*^0^) and after (*i*) toxin binding. Figure 4a–e show the calibration curves of the five aptasensors in a plot of ((*i* − *i*^0^)*/i*^0^ *%*) as a function of the logarithm of toxin concentration. The calibration curves show good linearity with an excellent coefficient of determination (R^2^) and low detection limits (LODs) for the detection of the five toxins. The MC-LR aptasensor was applicable in the range of 0.073–150 nM, where the linear regression was ((*i* − *i*^0^)*/i*^0^ *%*) = 37.05 + 11.79 Log MC-LR concentration (nM) and the correlation coefficient R^2^ was equal to 0.95905. For CYL, the obtained linearity was within the range of 0.018 to 200 nM, where the linear regression was ((*i* − *i*^0^)*/i*^0^ *%*) = 25.28 + 10.17 Log CYL concentration (nM) and R^2^ was equal to 0.99231. The linear range of anatoxin was from 0.018 nM to 200 nM, where the linear regression was ((*i* − *i*^0^)*/i*^0^ *%*) = 33.82 + 12.61 Log anatoxin concentration (nM) and R^2^ was equal to 0.98766. The linear correlation for saxitoxin was in the range of 0.018 to 200 nM, where the linear regression was ((*i* − *i*^0^)*/i*^0^ *%*) = 28.41 + 13.18 Log saxitoxin concentration (nM) and R^2^ was equal to 0.91701. Finally, the OA analytical range was from 0.018 to 200 nM with the equation ((*i* − *i*^0^)*/i*^0^ *%*) = 29.73 + 11.96 Log OA concentration (nM) and R^2^ = 0.98881.

The sensitivity of each aptasensor was calculated as 3σ/slope, where σ is the standard deviation of the blank samples. Excellent values of limit of detection were obtained at 0.0033, 0.0045, 0.0034, 0.0053 and 0.0048 nM for MC-LR, CYL, anatoxin-α, saxitoxin and OA, respectively. These results confirm the high sensitivity of our multiplexed aptasensor for cyanotoxin detection as the LODs are much lower than the maximum admissible levels established by the WHO (1 µg/L). This high sensitivity could be attributed to the large surface area provided by the gold nanoparticles electrografted on the carbon surface, which allowed the immobilization of large number of aptamer molecules whereby the general signal was consequently proportional to the bioreceptor loading on the surface. AuNPs act, thus, as nanoscale amplifiers that electrically communicate between bioreceptors and bulk electrode materials [33].

### 3.5. Selectivity, Reproducibility and Stability

**Figure 5:**
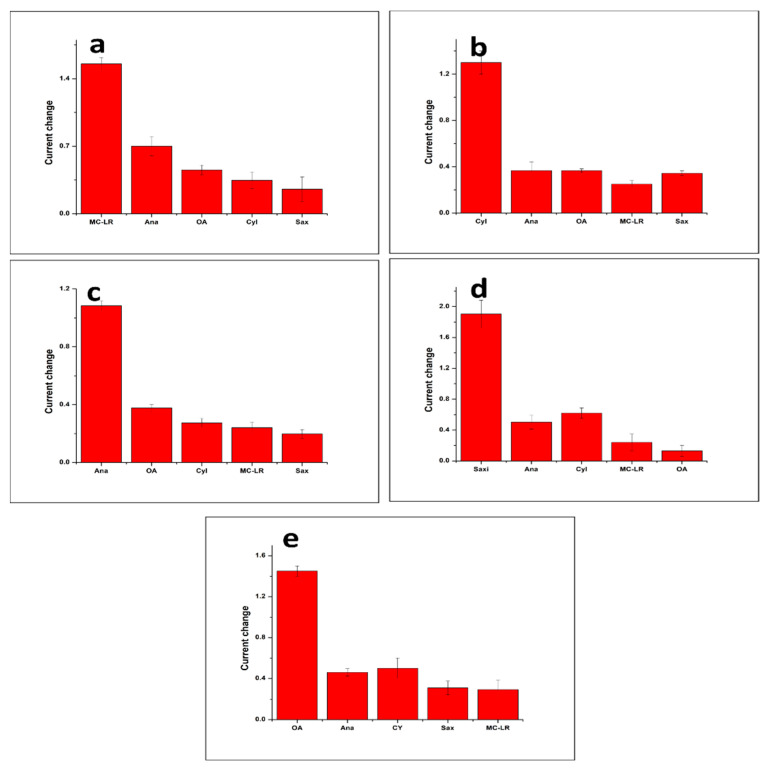
Selectivity studies of the multiplexed aptasensor. Cross reactivity of MC-LR (**a**), CYL (**b**), anatoxin-α (**c**), saxitoxin (**d**) and OA (**e**) aptasensors toward non-specific cyanotoxins (concentration of 100 nM). The current change was calculated as *i*^0^ − *i*, where *i*^0^ and *i* correspond to the current peak before and after incubation with the specific and non-specific cyanotoxins.

Since the proposed strategy aims to detect five cyanotoxins simultaneously, the cross-reactivity of each aptasensor of the array to the four other toxins should be assessed. For that, the five aptasensors were incubated individually with a fixed amount of the four non-specific toxins as well as the specific one. Then, the corresponding interaction between the aptamer and the toxin were monitored by SWV. The obtained electrochemical signals were used to calculate the current change (*i*^0^ − *i*) and compare the negative controls with the specific binding. Figure 5a–e show the bar charts of the multiplexed aptasensor array responses to the specific and non-specific analytes, corresponding to the MC-LR, CYL, anatoxin-α, saxitoxin and OA sensors, respectively. The figure reveals that the response of the aptasensors was much higher when incubated with the specific toxin, as compared to the slight increase obtained with the negative controls. These results confirm the specificity of each functionalized electrode of the microarray and the absence of cross-reactivity between them. Triplicate measurements were performed for all tests.

Besides selectivity, reproducibility is one of the key analytical characteristics of a performant biosensor. The multiplexed aptasensor displayed good reproducibility, expressed by relative standard deviations lower than 5% in the triplicate tests. Stability was also demonstrated; we observed that after two months of storage at 4 °C, the aptasensors had a negligible loss of activity.

### 3.6. Applicability of the Multiplexed Biosensor to Spiked Tap Water Samples

Finally, the feasibility of our multiplexed aptasensor for the simultaneous detection of cyanotoxins in freshwater was assessed. The microarray was incubated with tap water samples diluted in a binding buffer (1:50) spiked with known concentrations (0.58 nM) of each toxin (MC-LR, CYL, anatoxin-α, saxitoxin and OA). Then, the sensor’s responses were calculated as ((*i* − *i*^0^)*/i*^0^ *%*), which represents the percent of change in the current peak obtained with spiked and non-contaminated water samples. The responses were, subsequently, compared to that obtained in the buffer based on the calibration curves. As shown in Table 2, the measured concentrations were close to the added ones despite the presence of different ions in the water samples. Based on this comparison, the recovery percentages were calculated, showing good percentages varying from 90.16 to 109.45% with RSDs lower than 5%. RSDs were obtained by comparing the measured concentrations calculated for the three samples spiked with the same amount of toxins. These results demonstrate that the proposed biosensor can be successfully applied for synchronous detection of multiple cyanotoxins without matrix effects.

## 4. Conclusions

In summary, a novel multiplexed electrochemical platform for the detection of multiple cyanotoxins in freshwater was successfully developed for the first time. The platform is based on five specific aptamers, exhibiting high affinity for the targeted marine toxins, namely, MC-LR, CYL, anatoxin-α, saxitoxin and OA. The aptamers were individually immobilized on a microarray consisting of eight carbon electrodes modified with gold nanoparticles. The detection of complexation between the aptamers and their targets was based on the DNA conformational change, which induces a change in electron transfer that can be monitored electrochemically by SWV. The obtained results showed a wide linear range and high sensitivity for the five toxins, where the LODs were much lower than the limit concentrations allowed in drinking water. Importantly, no cross-reactivity was observed for the non-specific toxins, demonstrating the high selectivity of the proposed strategy. In parallel, negligible matrix effects were observed in the spiked tap water sample application experiments. The designed multiplexed aptasensor presents an excellent alternative to single aptasensors and immunosensors reported in the literature. In general, water is contaminated concurrently by different cyanotoxins and their simultaneous detection would reduce the time and cost of monitoring. Such a multiplexed detection platform holds great promise in the realm of analysis for various fields of applications, including medical diagnostics, environmental monitoring and food quality assessment.

## Figures and Tables

**Table 1:** Aptamer sequences used in the design of the multiplexed aptasensor.

Aptamer	Sequence	*Kd* (nM)	Ref.
MC-LR	5′-GGC GCC AAA CAG GAC CAC CAT GAC AAT TAC CCA TAC CAC CTC ATT ATG CCC CAT CTC CGC-3′	50	[26]
CYL	5′-GGC ATC AGG CAA CAA CCG ATG GTC CGG CCA CCC TAA CAA CCA GCC CAC CCA CCA CCC CGC CG-3′	88.78	[27]
Anatoxin-α	5′-TGG CGA CAA GAA GAC GTA CAA ACA CGC ACC AGG CCG GAG TGG AGT ATT CTG AGG TCG G-3′	81.3	[28]
Saxitoxin	5′-T TGA GGG TCG CAT CCC GTG GAA ACA GGT TCA TTG-3′	133	[29]
OA	5′-GGT CAC CAA CAA CAG GGA GCG CTA CGC GAA GGG TCA ATG TGA CGT CAT GCG GAT GTG TGG-3′	77	[30]

**Table 2:** Comparison of the multiplexed aptasensor performance in buffer and spiked tap water samples.

Cyanotoxin	Added Concentration (nM)	Measured Concentration (nM)	Recovery Percentage (%)	RSD (%)
MC-LR	0.58	0.63	109.45	4.19
CYL	0.58	0.52	90.16	3.21
Anatoxin-α	0.58	0.54	93.88	1.10
Saxitoxin	0.58	0.61	106.25	4.65
OA	0.58	0.53	91.5	2.23

## Data Availability

Data are contained within the article.
